# The social context of drinking and its association with days of heavy episodic drinking in the past month among a nationally representative sample of adolescents and young adults in the United States, 2021–23

**DOI:** 10.1093/alcalc/agag016

**Published:** 2026-03-30

**Authors:** Madison O Walsh, Matthew G Myers, Xiaoyu Liang, Kipling M Bohnert

**Affiliations:** Department of Epidemiology and Biostatistics, College of Human Medicine, Michigan State University, East Lansing, MI, USA; Department of Epidemiology and Biostatistics, College of Human Medicine, Michigan State University, East Lansing, MI, USA; Department of Epidemiology and Biostatistics, College of Human Medicine, Michigan State University, East Lansing, MI, USA; Department of Epidemiology and Biostatistics, College of Human Medicine, Michigan State University, East Lansing, MI, USA

**Keywords:** heavy episodic drinking, alcohol, young adult, social context, drinking alone

## Abstract

**Introduction:**

Prior research links solitary drinking under the age of 21 years, the legal drinking age in the USA, with adverse health later in life. This study examines associations between the social context of drinking (i.e. drinking alone vs with others) and the frequency of heavy episodic drinking (HED) days in the past 30 days among 12–20-year-olds in the USA.

**Materials and Methods:**

We aggregated National Survey on Drug Use and Health data from 2021 to 2023 for all individuals aged 12–20 years who drank alcohol in the past month (unweighted *n* = 6 261). The focal independent variable of interest was the social context of drinking in the past month, and the primary outcome was the categorical frequency of HED days in the past month. Weighted multinomial logistic regression was used to estimate associations, adjusting for sociodemographic factors.

**Results:**

Drinking alone, compared to drinking with others, was associated with a higher likelihood of HED on 20–30 days in the past month compared to 0 days in the adjusted model [relative risk ratio: 5.48, 95% confidence interval: 2.45, 12.25].

**Conclusions:**

Among this nationally representative sample of adolescents and young adults under the legal drinking age in the USA who drank alcohol in the past month, we found that drinking alone compared to drinking with others was associated with the highest frequency of HED days in the past month. This association was not observed across lower HED day frequency categories. Tailored interventions targeting individuals in this age range who drink alone may be necessary to help reduce high-frequency HED.

## Teaser

Among this nationally representative sample of 12–20-year-olds in the USA (unweighted *n* = 6 261), solitary drinking was associated with the highest frequency of HED days (20–30 days/month). No associations were detected for lower HED day categories in this analysis of 2021–23 National Survey on Drug Use and Health data. Targeted interventions for underage individuals who drink alone may be required to help reduce high-frequency HED.

## Introduction

The use of alcohol is a leading contributor to the global burden of disease, and alcohol remains one of the most used substances in the USA (Degenhardt et al. 2016, Murray et al. 2020). Among adolescents and young adults in the USA, 13.3% of individuals aged 12–20 years drank alcohol in the past month (SAMHSA 2024). Adolescence and young adulthood are critical periods in the development of lifelong health behaviors and alcohol use during these periods poses significant risks, including increased risks of both developing alcohol use disorder (AUD) and alcohol-related consequences (Nixon and McClain 2010). Risky alcohol use during these developmental periods, including heavy episodic drinking (HED), which is defined as four or more drinks on one occasion for females and five or more drinks on one occasion for males, is related to adverse health outcomes, including liver disease, injury, and death (Esser et al. 2024, National Institute on Alcohol Abuse and Alcoholism).

The age span of 12–20 years (adolescence through young adulthood) is a particularly important developmental period for alcohol-related risk behaviors. Across this span, individuals experience increasing autonomy in decision-making, transitions in educational environments, introduction to work settings, and shifts in social roles and peer influence. These changes make this a critical time for developing lifelong behaviors related to substance use. (Nixon and McClain 2010). Neurodevelopmentally, this period is characterized by ongoing maturation of the prefrontal cortex and heightened sensitivity to reward, both of which contribute to increased risk-taking behaviors, including substance use (Steinberg 2008).

The social context in which individuals drink can also change during adolescence and young adulthood, given the transition to a period of increased independence. Solitary substance use, in particular, may lead to riskier drinking behaviors due to the lack of peer influence, decreased social support, and more occasions of drinking compared to those who drink more socially (Creswell et al. 2015, 2022). Although pressure from peers may influence an individual to initiate alcohol use, prior research suggests that a lack of peer influence and societal norms regarding solitary substance use can increase the frequency and amount of alcohol used in a solitary setting (Gonzalez et al. 2009). Gonzalez et al. (2009) found that solitary HED in a college-aged (18–20 years) sample was associated with increased alcohol-related problems and was more strongly tied to coping motives than social motives, with coping motives related to the development of AUD. Creswell et al. (2022) also found that solitary alcohol use at ages 18 and 23–24 years was separately associated with greater odds of AUD symptoms at age 35, indicating that adolescent and young adult drinking behaviors may have lifelong impacts on health. Similarly, Patrick et al. (2023) found that HED during adolescence was associated with alcohol misuse in midlife, defined as AUD symptoms or high-intensity drinking (greater than 10 drinks/week), with stronger associations observed among males and White individuals (Patrick et al. 2023). The age range spanning early adolescence through young adulthood (12–20 years) encompasses substantial variation in psychosocial development, autonomy, and social context, while also sharing legal restrictions on alcohol access in the USA. Consequently, it is important to identify drinking patterns that emerge prior to legal access to alcohol which may signal elevated risk of harmful alcohol use. Transitions to the legal drinking age (i.e. 21 years) in the USA may shift the social context of drinking (e.g. ability to drink in bars). Similarly, although prior research has examined alcohol use frequency in relation to risky drinking and AUD, there is limited work investigating solitary drinking in relation to HED, which is particularly harmful to health (Centers for Disease Control and Prevention, n.d.). Additional studies, particularly those using national data that are generalizable to the US population, regarding the social context of drinking and drinking behaviors in this unique developmental period are needed.

Given the importance of the frequency of heavy alcohol use on health and development of risky drinking patterns, including AUD, this study focuses on the behavior of drinking alone and risky drinking in a critical developmental age span (White and Hingson 2019, De Goede et al. 2021). Using nationally representative data for 2021–23 from the National Survey on Drug Use and Health (NSDUH), this study examines potential links between the social context of drinking (i.e. drinking alone vs with others) and the categorical frequency of days of HED, defined as five or more drinks on the same occasion for males and four or more for females, in the past 30 days among 12–20-year-olds in the USA.

## Materials and Methods

For this analysis, we analyzed pooled data from the 2021 through 2023 NSDUH data. The NSDUH is an annually conducted and nationally representative survey that employs an independent, multistage area probability sampling design. The NSDUH samples the civilian noninstitutionalized population, aged 12 years or older, residing within the USA (unweighted *n* = 173 808).

Audio computer-assisted self-interviewing was used during in-person and web-based data collection to minimize biases associated with reporting sensitive behaviors. Additional details on data collection and NSDUH procedures are available elsewhere (SAMHSA 2022). The present analytic sample was restricted to individuals aged 12–20 years (unweighted *n* = 48 898) with past-month alcohol use (unweighted *n* = 6861) and without missing data for all the variables included in the analysis (unweighted *n*  = 6261). The Michigan State University Institutional Review Board has deemed the deidentified NSDUH data as non human subjects research.

The primary outcome of interest for the present study was the frequency of HED days in the past month, which was defined as four or more drinks on one occasion for females and five or more drinks on one occasion for males (NIAAA 2022). For this analysis, the count of HED days in the past month was categorized into four groups: 0, 1–2, 3–5, 6–19, and 20–30 days, following the recoding procedures of the NSDUH (SAMHSA 2024).

The main exposure of interest was the social context of the most recent drinking experience. Categorical responses from NSDUH consisted of drinking alone, with one other person, with more than one person, and 21+/never used alcohol in the past month. The survey questions specifically asked, “This last time you drank, were you alone or were you with one or more other people?”, with the responses “I was alone the last time I drank,” “I was with one other person the last time I drank,” or “I was with more than one other person the last time I drank.” For the purposes of this research question and due to the small cell size, the responses “with one other person” and “with more than one person” were aggregated into a single category. The final categories examined in this analysis were “drinking alone” and “drinking with others.”

The covariates used in this analysis were included in alignment with previous research (Creswell et al. 2022). Sex was classified as male or female using the IRSEX variable. Due to limitations in sample size, race/ethnicity was recorded from seven categories in the NEWRACE2 variable to “White” and “non-White.” Age category was classified from the CATAG7 variable in NSDUH into 12–15, 16–17, and 18–20 years*.* The age categories 12–13 and 14–15 years were collapsed due to small cell size. Household Income was classified from the INCOME variable in NSDUH into <$20 000, $20 000–$49 999, $50 000–$74 999, and >$75 000.

### Analysis

We first conducted bivariate analyses to estimate the prevalences of HED in the past month across the demographic factors of sex, race, age category, and household income. We present weighted percentages and 95% confidence intervals (CIs), and we used chi-square tests to assess potential group differences in HED categories. We fitted unadjusted and covariate-adjusted multinomial logistic regression models to estimate associations between the social context of drinking alone compared with others and the category of number of HED days in the past 30 days. We used multinomial logistic regression due to potential violations of the proportional odds assumption, which would limit the appropriateness of ordinal logistic regression for this outcome. Results are presented as relative risk ratios (RRRs) with 95% CIs. An RRR > 1 indicates a higher relative likelihood of being in a specific HED category compared with the reference category (0 HED days), while an RRR < 1 indicates a lower likelihood. All analyses incorporated NSDUH-created survey design variables (weight, stratification, and clustering) and the full sampling information to produce nationally representative estimates for 12–20 year olds in the USA with past-month alcohol use. As a sensitivity check, we fitted an additional multinomial logistic regression model examining the categorical frequency of alcohol use in the past 30 days (1–2, 3–5, 6–19, and 20–30 days) as the outcome to assess whether the association was consistent for solitary drinking with this less risky drinking behavior. We performed analyses using Stata software with complex survey commands (StataCorp, 2025, *Stata Statistical Software: Release 19*, College Station, TX: StataCorp LLC).

## Results

Table 1 describes the demographic characteristics of the present analytic sample of 12- to 20-year-olds who drank alcohol in the past month. Higher frequency HED day categories tended to be more prevalent among those who typically used alcohol with others, those aged 18–20 years, and those whose household incomes were >$75,000/year. Although not significant, White individuals had higher prevalences of HED days across all of the categories, compared to non-White individuals. Higher HED day categories were most prevalent among 18–20-year-olds compared with 12–15- and 16–17 –year-olds. HED was more prevalent among individuals with household incomes > $75,000 across all HED day categories. Overall, respondents more commonly drank with at least one other person (89.24%; 95% CI: 87.80, 90.68) than having most recently drank alone (10.76%; 95% CI: 9.32, 12.20).

**Table 1:** Demographic characteristics of 12–20-year-olds with past-month alcohol use, by category of past-month heavy episodic drinking days (unweighted *n* = 6261).

**Category of heavy episodic drinking days in the past month**							
	**Total sample unweighted *n* = 6261**	**0 days unweighted** ***n* = 2735**	**1–2 days unweighted** ***n* = 2174**	**3–5 days unweighted** ***n* = 843**	**6–19 days unweighted** ***n* = 452**	**20–30 days unweighted** ***n* = 57**	***P*-value**
**Social context of most recent alcohol use event^a^**							
Alone	10.76 (9.32, 12.20)	11.94 (10.22, 13.66)	9.44 (7.28, 11.60)	8.99 (5.09, 12.91)	8.569 (4.45, 12.69)	40.24 (13.15, 67.33)	**.0003^b^**
With others	89.24 (87.80, 9.68)	88.06 (86.34, 89.78)	90.56 (88.40, 92.72)	91.01 (87.09, 94.91)	91.43 (87.31, 95.55)	59.76 (32.67, 86.85)	
**Race**							
White	59.88 (57.54, 62.22)	57.19 (54.07, 6.31)	59.68 (56.05, 63.31)	62.47 (56.78, 68.16)	70.89 (62.33, 79.45)	69.93 (58.43, 100.00)	.1018
Non-White	40.12 (37.78, 42.46)	42.81 (39.69, 45.93)	40.32 (36.69, 43.95)	37.53 (31.84, 43.22)	29.11 (20.55, 37.67)	30.07 (4.57, 6.63)	
**Sex**							
Male	47.95 (45.75, 50.15)	47.69 (44.64, 5.74)	47.82 (43.57, 52.07)	43.14 (38.24, 48.04)	57.26 (49.49, 65.03)	57.74 (32.30, 83.18)	.115
Female	52.05 (49.85, 54.25)	52.31 (49.26, 55.36)	52.18 (47.93, 56.43)	56.86 (51.96, 61.76)	42.74 (34.97, 5.51)	42.26 (16.82, 67.70)	
**Age (years)**							
12–15	10.03 (8.83, 11.23)	12.12 (10.40, 13.84)	10.98 (8.92, 13.04)	5.11 (2.94, 7.28)	3.21 (1.00, 5.42)	1.28 (0.00, 2.89)	**.0000**
16–17	20.49 (18.84, 22.14)	20.99 (18.63, 23.35)	22.90 (19.88, 25.92)	18.96 (14.50, 23.42)	8.13 (5.43, 1.83)	24.38 (0.00, 5.30)	
18–20	69.48 (67.36, 71.60)	66.88 (64.19, 69.57)	66.13 (62.21, 7.05)	75.93 (70.74, 81.12)	88.66 (85.25, 92.07)	74.35 (48.48, 100.00)	
**Income ($)**							
<20 000	20.04 (17.95, 22.13)	16.65 (14.08, 19.22)	21.15 (18.19, 24.11)	24.97 (19.75, 30.19)	28.60 (21.19, 36.01)	5.54 (0.82, 1.26)	**.0049**
20 000–49 999	22.38 (20.34, 24.42)	21.41 (19.03, 23.79)	23.79 (20.28, 27.30)	22.80 (17.43, 28.17)	18.69 (12.33, 25.05)	36.14 (5.21, 67.07)	
50 000–74 999	12.01 (10.48, 13.54)	11.45 (9.51, 13.39)	11.67 (9.81, 13.53)	13.59 (8.85, 18.33)	11.96 (7.27, 16.65)	25.98 (0.00, 54.30)	
>75 000	45.56 (43.43, 47.69)	50.49 (47.07, 53.91)	43.39 (39.81, 46.97)	38.64 (33.62, 43.66)	40.76 (33.99, 47.53)	32.34 (10.52, 54.16)	

^a^Weighted percentages with 95% CIs are displayed.

^b^Bold indicates *P* < .05 using chi-squared test accounting for the complex survey design.

Table 2 displays the results from the adjusted multinomial logistic regression model estimating the association between the social context of drinking and HED days among 12–20–year-olds who drank in the past month. Drinking alone compared to drinking with others was associated with a higher likelihood of HED on 20–30 days in the past month than 0 days, both in unadjusted (RRR: 4.97, 95% CI: 1.63, 15.10; *P*-value: .003; not shown in a table) and adjusted models (RRR: 5.48, 95% CI: 2.45, 12.25; *P*-value: .000). Drinking alone was not significantly associated with other HED day categories (1–2, 3–5, and 6–19 days). In further comparison testing (i.e. changing the reference group to the other categories and estimating the model), drinking alone was significantly associated with HED on 20–30 days in the past month compared with each of the other categories of HED days in the past month.

**Table 2:** Adjusted multinomial logistic regression model estimating associations between the social context of drinking and the category of heavy episodic drinking days in the past month among 12–20-year-olds who drank in the past month (unweighted *n* = 6261).

	**Heavy episodic drinking days in past month**				
	0	**1–2** **RRR (95% CI)**	**3–5** **RRR (95% CI)**	**6–19** **RRR (95% CI)**	**20–30** **RRR (95% CI)**
**Adjusted (full model)**					
With others	Ref	Ref	Ref	Ref	Ref
Alone	Ref	0.77 (0.57, 1.03)	0.81 (0.58, 1.14)	0.79 (0.43, 1.44)	**5.48 (2.45, 12.25)**
**Race**					
White	Ref	Ref	Ref	Ref	Ref
Non-White	Ref	0.85 (0.69, 1.06)	**0.74 (0.56, 0.98)**	**0.52 (0.33, 0.83)**	0.42 (0.11, 1.54)
**Sex**					
Male	Ref	Ref	Ref	Ref	Ref
Female	Ref	0.96 (0.79, 1.15)	1.83 (0.93, 1.50)	**0.68 (0.47, 0.99)**	0.82 (0.27, 2.56)
**Age (years)**					
12–15	Ref	Ref	Ref	Ref	Ref
16–17	Ref	1.19 (0.90, 1.58)	**2.17 (1.32, 3.59)**	1.47 (0.64, 3.38)	**13.35 (2.55, 69.85)**
18–20	Ref	1.00, (0.77, 1.32)	**2.47 (1.48, 4.13)**	**4.43 (2.18, 9.00)**	**14.16 (3.26, 61.57)**
**Income ($)**					
<20 000	Ref	Ref	Ref	Ref	Ref
20 000–49 999	Ref	0.89 (0.67, 1.18)	0.79 (0.52, 1.21)	0.66 (0.39, 1.09)	**5.08 (1.64, 15.69)**
50 000–74 999	Ref	0.79 (0.55, 1.15)	0.86 (0.58, 1.29)	0.72 (0.38, 1.35)	**8.36 (1.31, 53.34)**
>74 999	Ref	**0.64 (0.48, 0.87)**	**0.54 (0.37, 0.78)**	**0.54 (0.36, 0.82)**	1.99 (0.68, 5.88)

Abbreviations: CI, confidence interval; RRR, relative risk ratio.

Bold indicates *P*-value < 0.05.

In addition, results from the adjusted multinomial logistic regression show that non-White individuals were less likely to drink 3–5 days (RRR: 0.74; 95% CI: 0.56, 0.98) and less likely to drink 6–19 days (RRR: 0.52; 95% CI: 0.33, 0.84) than 0 days. We did not find a significant difference between males and females in the highest HED frequency category. However, females were less likely than males to have 6–19 HED days (RRR: 0.68 95% CI: 0.47, 0.99), compared to 0 HED days. Older age was associated with higher likelihoods of more frequent HED. Compared with individuals aged 12–15 years, those aged 16–17 years were more likely to report 3–5 HED days (RRR: 2.17; 95% CI: 1.32, 3.59) and 20–30 days (RRR: 13.35; 95% CI: 2.55, 69.85), relative to 0 days. Individuals aged 18–20 years were also more likely to report HED on 3–5 days (RRR: 2.47; 95% CI: 1.48, 4.13), 6–19 days (RRR: 4.43; 95% CI: 2.18, 9.00), and 20–30 days (RRR: 14.16; 95% CI: 3.26, 61.57), relative to 0 days. No significant differences were observed for 1–2 HED days across age groups. Compared to those with incomes < $20 000, individuals with household incomes > $75 000 were less likely to report 1–2 days (RRR: 0.64; 95% CI: 0.48, 0.87), 3–5 days (RRR: 0.54; 95% CI: 0.37, 0.78), and 6–19 days (RRR: 0.54; 95% CI: 0.36, 0.82) of HED than 0 days. Individuals with incomes of $20 000–$49 999 were more likely to have 20–30 days of HED (RRR: 5.08; 95% CI: 1.64, 15.69). Similarly, individuals with income $50 000–$74 999 were more likely to have 20–30 HED days (RRR: 8.36; 95% CI: 1.31, 53.34) than those with income < $20 000.

In a sensitivity check among the analytic sample, we examined categories of frequency of alcohol use days in the past month. Solitary drinking was associated with the highest frequency drinking category (20–30 days). Namely, drinking alone compared to drinking with others was associated with a four-fold greater likelihood of drinking on 20–30 days in the past month compared to 1–2 days (RRR: 4.04; 95% CI: 1.94, 8.38; not shown in a table); no significant associations were observed for lower frequency drinking categories.

## Discussion

This study explored associations between the social context of drinking alcohol (i.e. drinking alone vs with one or more other person[s]) and the category of HED days in the past month among a nationally representative sample of emerging adults aged 12–20 years in the USA. The results showed that drinking alone was associated with a higher likelihood of HED on 20–30 days per month, the highest category of HED days, compared to any of the other categories.

The association between solitary drinking and number of HED days in the past month was associated only at the highest level of frequency (i.e. 20–30 days), and not associated with less frequent (1–2, 3–5, and 6–19 days) HED categories. It is important to note that solitary HED may not be a common behavior among all adolescents and young adults given that 10.76% of the total sample in this study drank alone the last time they drank. However, the finding that 40.24% of individuals reporting HED on 20–30 days in the past month drank alone on their most recent drinking occasion suggests a smaller high-risk subgroup with particularly concerning drinking patterns that may warrant further investigation and potential intervention.

These results align with prior research underscoring the risks associated with solitary substance use. Although drinking in a social environment is often tied to heavy drinking, solitary drinking may reflect a different underlying motivation of use that might encourage more risky drinking behaviors (Creswell et al. 2015, 2022). Coupled with our findings, this suggests that early solitary use of alcohol could signal a path toward riskier alcohol consumption behaviors; however, further longitudinal work in this area is needed to clarify the directionality and underlying mechanisms. The cross-sectional nature of these data prevents determination of whether the social context of alcohol drinking facilitates heavier consumption or whether individuals who engage in HED increasingly withdraw to solitary environments to avoid the stigma associated with HED. Understanding these pathways might have implications for intervention design, determining whether, e.g., efforts should focus on promoting social engagement to prevent HED or prioritize harm reduction and social reconnection for those already drinking heavily in isolation. Gonzalez et al. (2009) found that solitary HED in young adulthood was associated with increased alcohol-related problems and was more strongly tied to coping motives than social motives, suggesting that individuals who drink alone may be using alcohol to manage emotional distress, potentially placing them at greater risk for long-term consequences of increased risky drinking patterns and unhealthy alcohol use. Nonetheless, additional longitudinal research is needed in this area to determine whether patterns that develop during this age period continue and impact health outcomes through later life. The present study builds on prior work by presenting more recent and nationally representative estimates. Moreover, the results from this analysis show that solitary drinking is a notable marker of very frequent HED among adolescents and young adults, who are entering a more independent period in their lives yet cannot drink legally. The 12–20-year-old age span marks a unique developmental period in the USA characterized by substantial changes in autonomy, social environments, and decision-making contexts. Across this age range, individuals experience transitions such as increasing independence from caregivers, greater peer influence, and evolving social roles, whether via secondary school, postsecondary education, or entry into the workforce (Nixon and McClain 2010). This period is distinct from broader, older age groups that are typically studied (e.g. 21–25 years), as this age group captures underage drinkers in the USA who have varying degrees of independence. After the age of 21, the legal drinking age in the USA, the social context that an individual most often drinks in may naturally change to a more social environment due to the lack of legal restrictions. Nonetheless, 12–20-year-olds are unique and remain under the legal drinking age in the USA. Focusing on this underage period allows for a more nuanced examination of alcohol use patterns that emerge prior to legal drinking access and may signal elevated risk for the development of longer-term, high-risk drinking behaviors as well as other adverse health and social outcomes.

Consistent with other nationally representative estimates, non-White participants were less likely than White participants to engage in more frequent days of HED (Krieger et al. 2018). Although males showed slightly higher prevalences of number of HED days than females, the only significant difference between the sexes was among the 6–19 days category. In contrast, age was strongly associated with HED frequency, with older adolescents and young adults demonstrating higher likelihoods of more frequent HED compared with younger adolescents. These findings highlight age-related escalation in HED within the underage population and underscore the importance of considering developmental stage when examining patterns of high-risk alcohol use prior to the legal drinking age. Sensitivity analyses using past-month drinking frequency yielded similar patterns, with solitary drinking associated primarily with near-daily alcohol use, reinforcing the interpretation that solitary drinking may identify a subgroup of adolescents and young adults with especially concerning and persistent drinking patterns who may benefit from targeted intervention.

Although further research is needed, from a public health perspective, solitary drinking represents an important and potentially modifiable risk factor, suggesting multiple avenues for intervention. Our findings have several implications for potential prevention and intervention efforts. Healthcare providers and school health services could incorporate assessment of drinking contexts into routine alcohol screening to identify individuals who frequently drink alone. Prevention programs might expand beyond social drinking scenarios to explicitly address solitary drinking and its associated risks. Interventions that promote social connection, teach alternative coping strategies, and address underlying mental health concerns might also be particularly beneficial for emerging adults who drink alone. Given the association between solitary drinking and frequency of HED, future intervention research should examine whether targeting drinking contexts can effectively reduce alcohol-related harms in this population.

This study has several potential limitations that should be considered. First, the measure of the social context of alcohol use was based on respondents’ most recent drinking occasion, which may not fully represent their typical or habitual drinking patterns. Although this cross-sectional approach has inherent limitations in characterizing behavior, prior research shows that in a group of college-aged students, the social context of the most recent drinking event was indicative of typical overall drinking behavior (Armeli et al. 2014), providing support for the validity of this measure. This social context of drinking variable was also dichotomized due to sample size limitations. This could, e.g., obscure more nuanced differences, such as differences between small and large group drinking settings. Future studies with larger sample sizes and more detailed assessments of drinking contexts would help clarify whether different types of social drinking settings have differential associations with HED frequency patterns.

Similarly, collapsing the race/ethnicity categories into a binary variable due to small cell size limits the ability to understand disparities across more specific racial and ethnic subgroups. When stratifying by social context and examining HED outcomes, modeling race/ethnicity with seven separate categories provided by the NSDUH results in small cell sizes. However, we recognize that this binary categorization is oversimplistic and obscures important heterogeneity. Drinking patterns, cultural norms around alcohol use, and the social contexts in which drinking occurs vary considerably across Hispanic/Latino, Black/African American, Asian American, Native American, and other racial and ethnic groups. By collapsing these diverse populations into a single “Non-White” category, we are unable to examine these culturally specific patterns and may mask meaningful differences. The use of NSDUH’s standardized HED frequency categories provides consistency with national surveillance but also imposes limitations. Specifically, the upper categories (6–19 and 20–30 days) encompass wider ranges of drinking frequency, which may obscure finer distinctions in drinking behavior and mask differences between very frequent and near-daily heavy episodic drinkers. Future studies may benefit from more granular frequency measures, if available. The NSDUH also relies on self-report, which may be subject to recall bias. Another limitation of the NSDUH is that the sampling strategy does not include people living in institutionalized group quarters, i.e. correctional facilities, mental institutions, or nursing homes; therefore, the results from this analysis may not be generalizable to these settings.

Despite these limitations, this work provides novel evidence that solitary drinking is linked to high-frequency past-month HED behaviors among adolescents and young adults, a group predisposed to risky substance use (SAMHSA 2024). The findings from this study highlight the potential need for targeted prevention and intervention strategies that account for not only how much adolescents and young adults drink, but also with whom they drink. To develop effective prevention and intervention efforts, future research may explore the motivations behind solitary drinking in this age group. Effective prevention and intervention efforts may benefit from a more nuanced understanding behind the motivations for solitary drinking behaviors while also considering developmental and demographic factors.

Thus, drinking alone on the most recent drinking occasion is associated with the highest-frequency category of HED in the past month among emerging adults aged 12–20 years in the USA. This is a critical age period when individuals are becoming independent and are developing lifelong substance use patterns. Consequently, identifying and addressing solitary drinking behaviors in adolescents and young adults may be an important component of public health strategies to reduce the burden of alcohol-related harms.

## Data Availability

The datasets generated during this study are available at https://www.samhsa.gov/data/data-wecollect/nsduh-national-survey-drug-use-and-health/datafiles.
